# Impact of Pre-Infection COVID-19 Vaccination on the Incidence and Severity of Post-COVID Syndrome: A Systematic Review and Meta-Analysis

**DOI:** 10.3390/vaccines12020189

**Published:** 2024-02-12

**Authors:** Milena Adina Man, Daniela Rosca, Felix Bratosin, Ovidiu Fira-Mladinescu, Adrian Cosmin Ilie, Sonia-Roxana Burtic, Ariadna Petronela Fildan, Camelia Melania Fizedean, Adelina Maria Jianu, Rodica Anamaria Negrean, Monica Steluta Marc

**Affiliations:** 1 Department of Medical Sciences-Pulmonology, University of Medicine and Pharmacy, “Iuliu Hatieganu”, 400012 Cluj Napoca, Romania; adina.man@umfcluj.ro; 2 Doctoral School, “Victor Babes” University of Medicine and Pharmacy Timisoara, 300041 Timisoara, Romania; felix.bratosin@umft.ro (F.B.); dr.soniaburtic@umft.ro (S.-R.B.); 3 Discipline of Infectious Diseases, “Victor Babes” University of Medicine and Pharmacy Timisoara, 300041 Timisoara, Romania; 4 Center for Research and Innovation in Precision Medicine of Respiratory Diseases, “Victor Babes” University of Medicine and Pharmacy Timisoara, 300041 Timisoara, Romania; mladinescu@umft.ro (O.F.-M.); marc.monica@umft.ro (M.S.M.); 5 Discipline of Pulmonology, “Victor Babes” University of Medicine and Pharmacy Timisoara, 300041 Timisoara, Romania; 6 Department III Functional Sciences, Division of Public Health and Management, “Victor Babes” University of Medicine and Pharmacy Timisoara, 300041 Timisoara, Romania; ilie.adrian@umft.ro; 7 Department II, Discipline of Medical Communication, “Victor Babes” University of Medicine and Pharmacy, 300041 Timisoara, Romania; 8 Department of Pulmonology, Faculty of Medicine, “Ovidius” University of Constanta, 900470 Constanta, Romania; petronela.fildan@365.univ-ovidius.ro; 9 Methodological and Infectious Diseases Research Center, Department of Infectious Diseases, “Victor Babes” University of Medicine and Pharmacy, 300041 Timisoara, Romania; fizedean.camelia@umft.ro; 10 Department of Anatomy and Embriology, “Victor Babes” University of Medicine and Pharmacy, 300041 Timisoara, Romania; adelina.jianu@umft.ro; 11 Department of Physiology, Faculty of Medicine and Pharmacy, University of Oradea, 410073 Oradea, Romania

**Keywords:** COVID-19, vaccination, SARS-CoV-2, long-COVID, post-COVID-19 syndrome, coronavirus, infection

## Abstract

This systematic review critically evaluated the impact of a pre-infection COVID-19 vaccination on the incidence and severity of post-COVID-19 syndrome and aimed to assess the potential protective effect across different vaccines and patient demographics. This study hypothesized that vaccination before infection substantially reduces the risk and severity of post-COVID-19 syndrome. In October 2023, a comprehensive literature search was conducted across three databases, PubMed, Embase, and Scopus, focusing on studies published up to that date. Utilizing a wide array of keywords, the search strategy adhered to the PRISMA guidelines and was registered in the Open Science Framework. The inclusion criteria comprised studies focusing on patients with a breakthrough SARS-CoV-2 infection who developed post-COVID-19 syndrome. We included a total of 13 articles that met the inclusion criteria, analyzing more than 10 million patients with a mean age of 50.6 years, showing that the incidence of intensive care unit (ICU) admissions post-vaccination was as low as 2.4%, with a significant reduction in mortality risk (OR 0.66, 95% CI 0.58–0.74). The prevalence of post-COVID-19 syndrome symptoms was lower in vaccinated individuals (9.5%) compared to unvaccinated (14.6%), with a notable decrease in activity-limiting symptoms (adjusted OR 0.59, 95% CI 0.48–0.73). Vaccinated patients also showed a quicker recovery and return to work (HR 1.37, 95% CI 1.04–1.79). The pooled odds ratio of 0.77 indicates that vaccination is associated with a 23% reduction in the risk of developing post-COVID-19 syndrome (95% CI 0.75–0.79). Despite the protective effects observed, a substantial heterogeneity among the studies was noted. In conclusion, a pre-infection COVID-19 vaccination is associated with a significant reduction in the risk and severity of post-COVID-19 syndrome. However, the observed heterogeneity across studies suggests a need for further research with standardized methods to fully comprehend vaccine efficacy against long COVID.

## 1. Introduction

The ongoing coronavirus disease 2019 (COVID-19) pandemic, caused by the novel severe acute respiratory syndrome coronavirus 2 (SARS-CoV-2), has led to significant global morbidity and mortality, posing substantial public health challenges [1,2,3,4,5]. As of November 2023, over 13 billion doses of COVID-19 vaccines have been administered worldwide, representing a monumental effort in the history of medicine [6,7,8]. While these vaccines have been essential in reducing the incidence and severity of acute COVID-19 infections [9,10,11], there is an emerging concern regarding their role in influencing the long-term sequelae of the infection, often considered as the post-COVID-19 syndrome or long COVID [12].

Post-COVID-19 syndrome, encompassing a wide range of symptoms persisting after the acute phase of infection, is reported to affect approximately 5–30% of COVID-19 survivors [13,14]. This wide range in prevalence is attributed to differences in study methodologies, symptom reporting, and patient follow-up. In this context, understanding the impact of pre-infection COVID-19 vaccination on the incidence and severity of post-COVID-19 syndrome becomes crucial.

Recent studies have begun to shed light on this aspect. For instance, a large-scale study in Sweden involving approximately 600,000 individuals found that of almost 300 thousand vaccinated COVID-19 patients, only 0.4% were diagnosed with post-COVID-19 syndrome during follow-up, compared to 1.4% of the 290,030 unvaccinated patients [15]. This suggests a significant protective effect of vaccination, with an overall vaccine effectiveness in more than half of the patients against post-COVID-19 syndrome. Notably, the effectiveness was observed to increase with the number of vaccine doses received [16].

Given these findings, this systematic review aims to synthesize current evidence on the impact of pre-infection COVID-19 vaccination on the incidence of post-COVID-19 syndrome and to evaluate the extent of protective effect across different vaccines and patient demographics. The hypothesis is that COVID-19 vaccination before infection significantly reduces the risk of developing post-COVID-19 syndrome and its severity. This synthesis of evidence will provide valuable insights for clinicians, public health policymakers, and the broader medical community, informing future strategies to mitigate the long-term impacts of COVID-19 sequelae.

## 2. Materials and Methods

### 2.1. Protocol and Registration

The systematic review search was carried out in October 2023 across three electronic databases, including PubMed, Embase, and Scopus, focusing on literature published from 2020, since the inception of the COVID-19 pandemic, up to October 2023. The search was extensive, employing a wide array of keywords and phrases, comprising “COVID-19 Vaccine,” “SARS-CoV-2,” “Post-COVID-19 Syndrome,” “Vaccination Prior to Infection,” “Vaccine Efficacy,” “Postacute COVID-19 Sequelae,” “Long COVID,” “Vaccine-Induced Immunity,” “Viral Diseases-Prevention and Control,” and “Post-Viral Sequelae.” The developed search strategy was intricate, combining these terms in various configurations to ensure a thorough retrieval of relevant literature as follows: (“COVID-19 Vaccines” OR “SARS-CoV-2 Immunization”) AND (“Post-COVID-19 Syndrome” OR “Long COVID” OR “Chronic SARS-CoV-2 Syndrome”) AND (“Pre-Infection Vaccination” OR “Vaccine Efficacy” OR “Viral Prevention and Control”). This review was conducted in alignment with the Preferred Reporting Items for Systematic Reviews and Meta-Analyses (PRISMA) guidelines and with the International Prospective Register of Systematic Reviews (PROSPERO), establishing a framework for methodical analysis and ensuring transparency and rigor in our approach [17,18]. This review was registered in the Open Science Framework, with the registration code osf.io/yqkwd.

### 2.2. Eligibility Criteria and Definitions

Only English-language journal articles were considered, focusing on the most recent developments in COVID-19 vaccination and its impact on post-COVID-19 syndrome. The initial stage of selection involved removing duplicate entries, followed by a detailed evaluation of abstracts by two independent researchers to ensure relevance to our research questions. This process included cross-referencing using bibliographies from selected full-text articles. A third researcher was involved in clarifying conflicts in the decision-making process.

Inclusion criteria included the following: (1) studies that investigated the correlation between COVID-19 vaccination before SARS-CoV-2 infection and the incidence or severity of post-COVID-19 syndrome; (2) studies that included clinical outcome measures such as the prevalence, duration, and severity of post-COVID-19 syndrome symptoms; (3) studies that provided explicit details on the methodology for assessing the vaccination status and timing relative to COVID-19 infection; and (4) a precise description of how the post-COVID-19 syndrome, also known as long-COVID was assessed. Conversely, exclusion criteria encompassed the following: (1) studies that analyzed vaccinated patients after SARS-CoV-2 infection; (2) studies that included patients without a defined diagnosis of COVID-19; (3) studies not reporting clinical outcomes data; and (4) non-peer-reviewed articles, case reports, case-series, in-vitro studies, conference proceedings, general reviews, commentaries, and editorial letters. This approach ensured a comprehensive and relevant collection of data for analysis in our systematic review.

Long-COVID, also known as post-COVID-19 syndrome, is a term that broadly defines the signs, symptoms, and conditions that continue or develop after the acute phase of a COVID-19 infection. This definition was developed collaboratively by the Department of Health and Human Services (HHS) and the Center for Disease Control (CDC) [19]. It encompasses a wide range of symptoms and conditions that some individuals experience for four or more weeks following an initial infection by SARS-CoV-2, the virus responsible for COVID-19. The World Health Organization (WHO) further specifies that post-COVID-19 syndrome, or long COVID, can affect anyone exposed to SARS-CoV-2, regardless of the age or severity of the original symptoms [20]. According to the WHO, it is defined as the continuation or development of new symptoms 3 months after the initial SARS-CoV-2 infection, with these symptoms persisting for at least 2 months and not attributable to an alternative diagnosis.

### 2.3. Data Collection Process

The initial exploration of the databases revealed a total of 2242 publications, out of which 355 articles were examined after eliminating the majority based on title and abstract screening. A total of 172 studies were found to be duplicates and were subsequently eliminated. An additional 170 articles were excluded after a full text read due to a lack of data or not matching the inclusion criteria. This phase was conducted collaboratively by two authors. Discrepancies during this stage were reconciled by a third author, ensuring the precision and impartiality of the review process.

**Figure 1:**
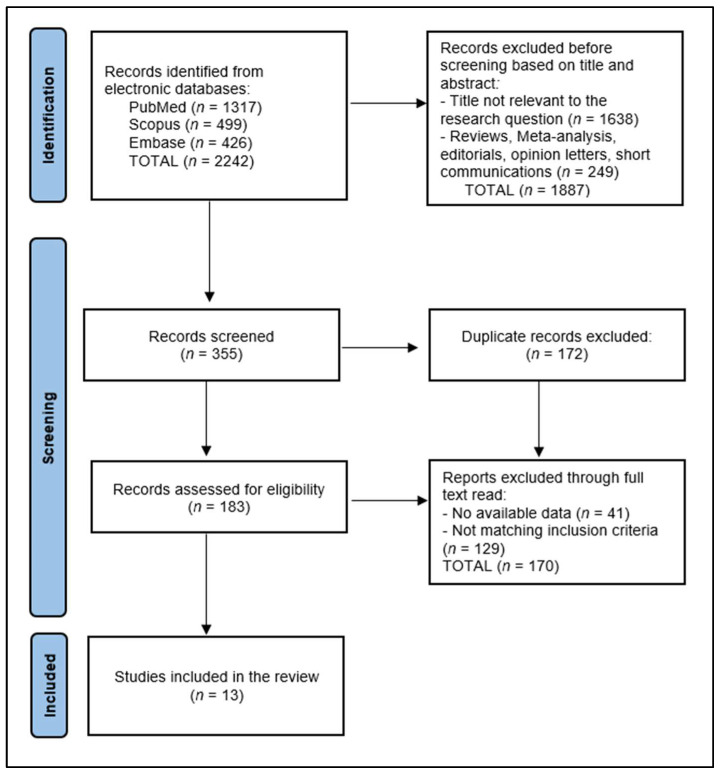
PRISMA Flow Diagram.

Ultimately, 13 articles satisfied the selection criteria and were incorporated into the review. The data extraction phase, undertaken by two investigators (M.A.M. and M.S.M.), involved a comprehensive collection and analysis of data pertaining to the study design, demographic characteristics of the participants, the type of vaccine and its dosing regimen, the timing of vaccination in relation to COVID-19 infection, and outcomes associated with post-COVID-19 syndrome, elements that are described in Figure 1.

### 2.4. Risk of Bias and Quality Assessment

In assessing the quality of the studies, the Newcastle–Ottawa Scale was utilized for cohort studies, while the Cochrane Collaboration’s tool was applied to randomized trials. Two independent researchers conducted evaluations of each study, assigning them quality ratings of low, medium, or high, providing a neutral appraisal of the selected publications, which served as a foundation for our systematic review.

**Figure 2:**
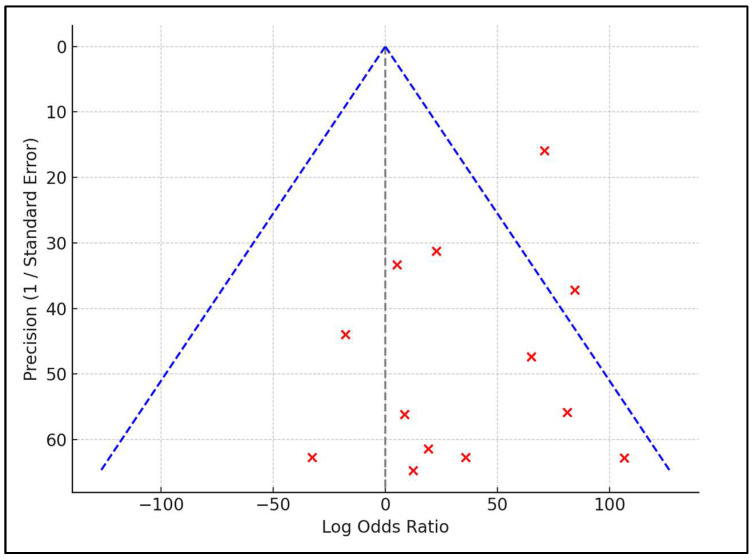
Funnel plot for publication bias.

To examine publication bias, a funnel plot was constructed, juxtaposing effect sizes with their respective standard errors. The plot symmetry was further scrutinized using Egger’s regression test, opting for a *p*-value of less than 0.05 to indicate a significant bias in publication. In addition, a sensitivity analysis was undertaken to ascertain the robustness of our results in order to help determine the impact of single studies on the aggregate outcomes, as depicted in Figure 2.

## 3. Results

### 3.1. Study Characteristics

The systematic review included thirteen studies [21,22,23,24,25,26,27,28,29,30,31,32,33] focusing on the impact of pre-infection COVID-19 vaccination on the incidence and severity of post-COVID-19 syndrome, as detailed in Table 1. The studies spanned five countries, emphasizing the global interest in understanding the role of the COVID-19 vaccination in mitigating long-term COVID-19 effects. The United States was the most represented country (38.5%), contributing five studies [21,25,28,30,33]. The United Kingdom followed with four studies [22,23,29,31], while Italy, Switzerland, Turkey, and the Netherlands each contributed one study [24,26,27,32], respectively.

In terms of publication year, six studies (46.1%) were published in 2023 [25,26,27,30,32,33] and seven in 2022 (53.9%) [21,22,23,24,28,29,31]. Regarding study design, the majority of the studies, nine in total (69.1%), utilized a retrospective cohort approach [21,23,24,26,28,29,31,33,34]. The remaining four studies employed a prospective cohort design [22,27,30,32], indicating a forward-looking approach to tracking the post-vaccination outcomes. The quality of these studies varied, with six studies rated as ‘High’ [22,25,27,28,30,32] and seven as ‘Medium’ [21,23,24,26,29,31,33].

### 3.2. Characteristics of Patients

The total number of patients across all studies was notably high, ranging from small cohorts, like Mohr et al. [30] with 419 patients, to extensive ones, like Meza-Torres et al. [29] with over 7.3 million patients; the total count was 10,147,451 patients with a breakthrough infection analyzed in these studies. Sex and gender distribution varied across studies, with some showing a higher representation of females, like Mohr et al. [30] (84.0% female) and Brannock et al. [25] (clinic-based: 65.0% female), while others, like Al-Aly et al. [21] and Ioannou et al. [28], had predominantly male participants (91.0% and 89.1% male, respectively). The average percentage of female participants across the studies was about 45.9%. 

The age of the participants also showed a wide diversity, with an average age across the included studies of 50.6 years. Studies like Azzolini et al. [24], with a mean age of 44.3 years, and Ballouz et al. [26], with a mean age of 48 years, focused on relatively younger populations, while Al-Aly et al. [21] had an older cohort with a mean age of 67 years. The comparison groups in these studies were primarily matched unvaccinated patients with COVID-19, providing a direct contrast to assess the impact of pre-infection vaccination. For example, Al-Aly et al. [21], Ayoubkhani et al. [23], and Ioannou et al. [28] used matched unvaccinated patients as their comparison group. Antonelli et al. [22] took a more nuanced approach, matching controls by post-vaccination test, healthcare worker status, and sex, which could offer more precise insights into the vaccine’s effects, as presented in Table 2.

### 3.3. COVID-19 Vaccination Characteristics

The type of vaccine administered was diverse across the studies. For example, Al-Aly et al. [21], Brannock et al. [25], Taquet et al. [31], and van der Maaden et al. [32] reported using either one dose of Janssen or two doses of Pfizer/Moderna vaccines. In contrast, Azzolini et al. [24] used the BNT162b2 vaccine for all three doses. Emecen et al. [27] provided insights into the use of CoronaVac and BNT162b2 vaccines, showcasing the variety of vaccine types used globally.

The number of vaccine doses varied from one to three, indicating different vaccination strategies. Ballouz et al. [26] presented a range from one to three doses, reflecting the evolving nature of vaccine administration protocols. The timing until a breakthrough infection occurred also varied considerably. Antonelli et al. [22] reported a mean of 73 days for the first case and 51 days for the second case post-vaccination, while Ballouz et al. [26] noted that the majority of breakthrough infections (77.6%) occurred more than six months after vaccination.

The follow-up periods in these studies were diverse, ranging from one month, as in Antonelli et al. [22] and Azzolini et al. [24], to up to eight months, as reported by Ioannou et al. [28]. This variance in follow-up duration provided a broad spectrum for observing post-vaccination effects. Notably, there were gaps in reported data for some studies. For instance, Meza-Torres et al. [29] and Zisis et al. [33] did not report specific vaccine types or other detailed vaccination characteristics, as presented in Table 3. 

### 3.4. Analysis of Outcomes

Table 4 highlights the varied impact of COVID-19 vaccination on reducing complications and the risk of post-COVID-19 syndrome. For instance, Al-Aly et al. [21] reported ICU admissions at 2.4%, with a post-COVID-19 syndrome risk odds ratio (OR) of 0.82 (0.80–0.85) and a significantly lower mortality risk at 0.66 (0.58–0.74), indicating that vaccination was associated with reduced severity of COVID-19 outcomes and a lower risk of post-COVID-19 syndrome. Antonelli et al. [22] focused on frail older adults, showing a significant reduction in hospitalization rates post-vaccination (23% post-first dose, 6% post-second dose). They also reported an increased risk of frailty in older adults post-first dose (OR 1.93, 95% CI 1.50–2.48) and an association with deprivation post-first dose (OR 1.11, 95% CI 1.01–1.23 for high deprivation). 

Ayoubkhani et al. [23] presented a comparison between double-vaccinated individuals and unvaccinated ones, with long COVID symptoms being 9.5% in the former group versus 14.6% in the latter. The adjusted odds ratio (aOR) for activity-limiting symptoms was 0.59 (95% CI, 0.48–0.73), indicating a protective effect of vaccination against severe post-COVID-19 syndrome symptoms. Azzolini et al. [24] provided a detailed breakdown of post-COVID-19 syndrome prevalence across different waves of the pandemic, with an overall prevalence of 31.0% and a significant reduction in risk (OR, 0.29). They also identified higher risks associated with older age, allergies, and more comorbidities.

**Figure 3:**
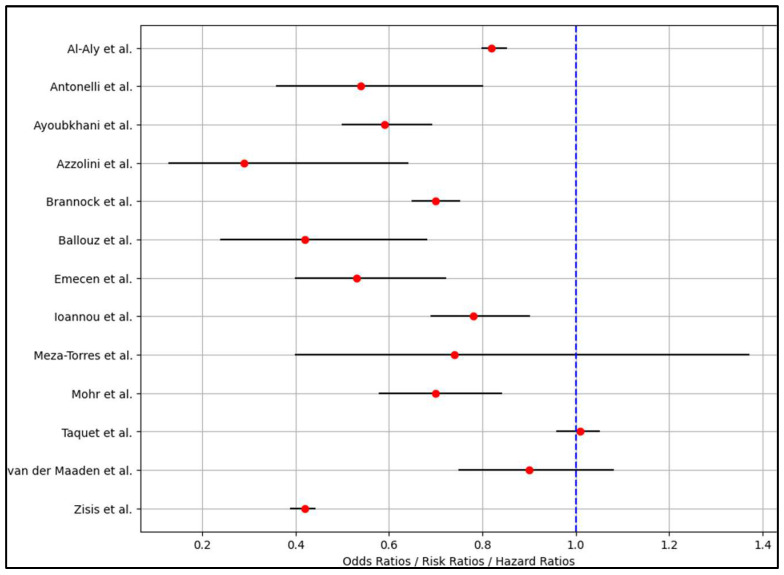
Forest plot analysis of the risk for post-COVID-19 syndrome among the analyzed studies (List of included references [21,22,23,24,25,26,27,28,29,30,31,32,33]).

Ballouz et al. [26] noted a variation in post-COVID-19 syndrome prevalence based on the type of SARS-CoV-2 variant, with the lowest prevalence after Omicron infection (13.1%). The study found a substantial absolute risk reduction in long COVID-related symptoms across variants. Other studies, such as Ioannou et al. [28] and Meza-Torres et al. [29], reported post-COVID-19 syndrome prevalence and associated risks, including higher odds of hospitalization and community or hospital infection. For example, Ioannou et al. [28] reported an adjusted odds ratio (AOR) of 2.60 for hospitalization and 2.46 for mechanical ventilation, suggesting that while vaccination reduces the risk of post-COVID-19 syndrome, certain risks remain elevated, as seen in Table 4 and Figure 3.

A meta-analysis of the studies assessing the impact of pre-infection COVID-19 vaccination on post-COVID-19 syndrome revealed a pooled odds ratio of 0.77, indicating that vaccination is associated with a 23% reduction in the risk of developing post-COVID-19 syndrome, with a high level of precision as evidenced by a narrow 95% confidence interval ranging from 0.75 to 0.79. However, the analysis also uncovered a significant level of heterogeneity among the studies, as indicated by an I^2^ statistic of nearly 98% and a Q statistic of about 586.81 with 12 degrees of freedom. 

## 4. Discussion

This systematic review of thirteen studies offers a comprehensive overview of the impact of pre-infection COVID-19 vaccination on the incidence and severity of post-COVID-19 syndrome. The studies, conducted across five countries, suggest a global consensus on the importance of understanding the long-term effects of COVID-19 and the role of vaccination in mitigating these effects. The diverse geographical distribution of the studies, with a predominant representation from the United States and the United Kingdom, underlines the widespread concern and research efforts directed towards managing the pandemic and its aftermath.

In addressing the evolving definitions of long COVID, also known as post-acute sequelae of COVID-19 (PASC), it is pertinent to consider the time frame of the studies reviewed, predominantly from 2022 and 2023, reflecting the delayed recognition and characterization of post-acute sequelae of SARS-CoV-2 infection, a phenomenon not clearly defined in the pandemic’s initial years, 2020 and 2021. The nascent nature of long COVID during this period led to a lack of consensus on its clinical criteria, influencing the research focus and outcomes. 

It is important to note the high number of individuals that were analyzed by this review: over 10 million COVID-19 patients with a breakthrough infection, with approximately equal proportions of men and women. However, the significant representation of females in some studies [21,28] contrasts with the predominantly male cohorts in others [30], suggesting potential gender-based differences in post-COVID-19 syndrome outcomes or healthcare-seeking behaviors. The average age of participants, at 50.6 years, indicates that the adult population is the most commonly affected by breakthrough infections and the development of post-COVID-19 syndrome, with a higher risk for severe COVID-19 complications. This demographic focus is essential for understanding the vaccine’s effectiveness across different age groups and for identifying particularly vulnerable populations.

The analysis of outcomes presents a compelling picture of the vaccine’s effectiveness in reducing the severity of COVID-19 and the risk of developing post-COVID-19 syndrome. Studies like Al-Aly et al. [21] and Antonelli et al. [22] demonstrate a clear reduction in ICU admissions, mortality, and hospitalization rates among vaccinated individuals. The protective effect against activity-limiting post-COVID-19 syndrome symptoms, as reported by Ayoubkhani et al. [23], further underscores the vaccine’s role in mitigating the impact of the disease. However, the presence of increased risks in specific cohorts, such as the older adults and those with comorbidities, as identified in studies like Azzolini et al. [24], indicates that while vaccination significantly reduces risks, it does not eliminate them.

The relationship between the severity of the initial COVID-19 infection and the subsequent development of post-COVID-19 syndrome has been a focal point in recent studies discussing the influence of age, female gender, and pre-existing health conditions [34,35]. Notably, the severity of the initial infection plays a critical role in this context [36], as various studies have established a link between the severity of the initial infection and both the frequency and duration of long-term sequelae [37,38]; they showed that patients who were hospitalized or admitted to the ICU were significantly more likely to experience post-COVID-19 syndrome, with risks increasing threefold and fivefold, respectively [39]. Moreover, patients who required supplemental oxygen or mechanical ventilation showed a higher propensity for persistent CT abnormalities at a one-year follow-up compared to those who did not require such interventions [40]. Given these findings, the role of vaccination, known for its efficacy in mitigating the severity of COVID-19 [41,42], becomes crucial, suggesting that vaccinated individuals might have a lower incidence of post-COVID-19 syndrome compared to those who are unvaccinated.

Furthermore, multicenter observational studies comparing vaccinated COVID-19 patients to unvaccinated ones, while controlling for known risk factors for severe infection, found a reduced incidence of post-COVID-19 syndrome among the vaccinated group over follow-up periods ranging from 28 to 180 days [31,33]. Intriguingly, other studies have noted that patients who received two doses of the vaccine were more likely to have pre-existing comorbidities compared to those who were either unvaccinated or had only one dose [21,22]. These comorbidities, such as asthma, diabetes, obesity, and immunosuppressive conditions, have been associated with a heightened risk of post-COVID-19 syndrome [43,44]. Therefore, our observation that patients with two-dose vaccinations, who also tend to have more pre-existing comorbidities, exhibited lower incidences of long COVID, further emphasizes the protective impact of vaccination against acute SARS-CoV-2 infection.

In contrast to our study, which focused on the effects of vaccination before COVID-19 infection, other research exploring the impact of vaccination post-infection, particularly in individuals with post-COVID-19 syndrome, has been more contentious. However, the studies we identified present a reassuringly consistent picture of vaccination being protective even after infection. Two specific studies examining remission [45] and recovery [46] from long COVID demonstrated promising results. They reported the odds of not recovering from long COVID when patients were vaccinated post-infection as 0.51 (95% confidence interval from 0.32 to 0.81) and 0.64 (0.17 to 2.33), respectively. This indicates a significant protective effect of vaccination even after the onset of COVID-19, suggesting potential benefits in terms of reducing the duration or severity of long COVID symptoms.

Nevertheless, although all these studies focused on the impact of COVID-19 on post-COVID-19 syndrome, there is still no generally accepted definition for this condition. Previously, standardized criteria for diagnosing post-COVID-19 conditions were lacking. A systematic review found that 65% of studies did not align with definitions from the CDC, NICE, or WHO [47], impacting the comparability of research. These studies varied in symptoms and durations for diagnosing post-COVID-19 syndrome conditions, frequently noting fatigue, muscle pain, anxiety, memory issues, and shortness of breath [48,49]. A separate study showed that COVID-19 survivors generally improved over time, with most returning to work within two years, but their health remained lower than the general population’s after this period [48]. The CDC describes post-COVID-19 syndrome symptoms as lasting over four weeks and sometimes resolving and recurring [50], differing from the WHO’s definition of symptoms appearing within three months of infection and persisting for two months [19]. A more consistent definition is crucial for understanding the prevalence of post-COVID-19 syndrome and vaccine efficacy against it.

Another important study by Thaweethai et al. is pivotal in establishing a symptom-based framework to identify PASC cases [51]. Their analysis of the RECOVER adult cohort highlighted 37 symptoms significantly more prevalent in SARS-CoV-2-infected individuals 6 months post-infection compared to uninfected individuals. This symptom-based approach provides a preliminary yet essential step in defining PASC as a distinct clinical condition. The identification of symptoms across multiple pathophysiological domains underscores the complex and multifaceted nature of PASC, resonating with the findings of our study, which also observed a wide range of post-infection sequelae in vaccinated individuals.

The implications of Thaweethai et al.’s findings for our study are manifold. Firstly, the symptom-based criteria they propose can be instrumental in refining the classification of PASC cases in future research, including studies like ours that investigate the impact of vaccination on long COVID outcomes. Secondly, the emphasis on iterative refinement incorporating clinical features aligns with the suggestions in our study to consider the variability in study designs and vaccine procedures. Our study, in conjunction with Thaweethai et al.’s framework, can aid in developing a more nuanced and clinically actionable definition of post-COVID-19 syndrome.

Our study’s findings must be considered in light of the diverse and evolving nature of the post-COVID-19 syndrome. The distinction between post-viral fatigue and immune-mediated damage due to spike protein interaction is crucial in understanding the range of post-COVID-19 syndrome manifestations. Furthermore, the emerging concept of post-vaccine post-COVID-19 syndrome adds another layer of complexity [52]. Our analysis suggests that different outcomes are likely based on these underlying mechanisms, with implications for both male and female populations who may experience post-COVID-19 syndrome differently. This underscores the need for ongoing research and nuanced clinical approaches in managing post-COVID-19 conditions.

This study, while thorough in approach, had certain limitations. The focus was solely on pre-infection vaccination studies, excluding post-infection vaccination research, which could provide additional insights into post-COVID-19 syndrome management. Variability in study designs, patient demographics, and the broad range of symptoms defining post-COVID-19 conditions could affect the robustness and generalizability of the findings. The varied approaches to vaccination, including the type of vaccine used and the number of doses administered, offer a comprehensive perspective on global vaccination strategies. The absence of specific vaccine details in some studies, however, points to a gap in data reporting that could impact the comprehensiveness and comparability of the findings. Moreover, the substantial heterogeneity uncovered by the meta-analysis suggests that the included studies vary greatly in factors such as population characteristics, types of vaccines used, or study methodologies. Such high variability underscores the need for caution in interpreting the pooled results and suggests that further exploration into the sources of this heterogeneity would be beneficial for a more nuanced understanding of the impact of COVID-19 vaccinations on post-COVID-19 conditions.

In addressing this study’s limitations, we must acknowledge the evolving definition of post-COVID-19 syndrome and its implications for our analysis. Early studies included in our review may have utilized differing criteria for post-COVID-19 syndrome, reflecting the ongoing development of understanding in this area. Additionally, our analysis could not account for potential immune damage due to T-cell responses against spike proteins or spike protein toxicity, which may manifest in various symptoms, including cardiac and neurological effects [53]. These factors underscore the complexity of post-COVID-19 syndrome and the need for caution in interpreting the study outcomes.

## 5. Conclusions

This systematic review comprehensively explored the impact of pre-infection COVID-19 vaccination on post-COVID-19 syndrome. The findings indicate that COVID-19 vaccines play a crucial role in mitigating the severity and incidence of post-COVID-19 syndrome, showcasing a notable protective effect. However, the definitive impact of vaccines in preventing or treating long COVID remains unclear due to the variability in study methodologies and definitions of post-COVID-19 syndrome. Similarly, it is unclear how long before SARS-CoV-2 infection the COVID-19 vaccines prevent post-COVID syndrome symptoms, if there is a significant difference between the type of vaccines and the number of vaccines administered, or if the protection for post-COVID-19 syndrome remains if the vaccines are administered after acute COVID-19. 

## Figures and Tables

**Table 1:** Study characteristics.

Reference Number and First Author	Country	Publication Year	Study Design	Study Quality
1 [21] Al-Aly et al.	United States	2022	Retrospective cohort	Medium
2 [22] Antonelli et al.	United Kingdom	2022	Prospective cohort	High
3 [23] Ayoubkhani et al.	United Kingdom	2022	Retrospective cohort	Medium
4 [24] Azzolini et al.	Italy	2022	Retrospective cohort	Medium
5 [25] Brannock et al.	United States	2023	Retrospective cohort	High
6 [26] Ballouz et al.	Switzerland	2023	Retrospective cohort	Medium
7 [27] Emecen et al.	Turkey	2023	Prospective cohort	High
8 [28] Ioannou et al.	United States	2022	Retrospective cohort	High
9 [29] Meza-Torres et al.	United Kingdom	2022	Retrospective cohort	Medium
10 [30] Mohr et al.	United States	2023	Prospective cohort	High
11 [31] Taquet et al.	United Kingdom	2022	Retrospective cohort	Medium
12 [32] van der Maaden et al.	Netherlands	2023	Prospective cohort	High
13 [33] Zisis et al.	United States	2022	Retrospective cohort	Medium

List of included references [21,22,23,24,25,26,27,28,29,30,31,32,33].

**Table 2:** Characteristics of patients.

Reference Number and First Author	Number of Patients	Sex/Gender	Age (Mean/Median)	Comparison Group
1 [21] Al-Aly et al.	33,940 vaccinated	91.0% Male; 9.0% Female	67.0 years (median)	Matched unvaccinated patients
2 [22] Antonelli et al.	1,240,009 (cases 1: first dose), 971,504 (cases 2: second dose)	Cases 1: 62.5% Female, 37.5% Male; Cases 2: 61.2% Female, 38.8% Male	Cases 1: 50.2 years (mean); Cases 2: 52.9 years (mean)	Controls matched by post-vaccination test, healthcare worker status, sex
3 [23] Ayoubkhani et al.	3333 double-vaccinated, 3090 matched unvaccinated	NR	Double-vaccinated: 49 years (mean); Unvaccinated: 47 years (mean)	Matched unvaccinated patients with COVID-19
4 [24] Azzolini et al.	739 (29% of 2560 participants had COVID-19)	32.7% Female, 26.1% Male (among COVID-19 cases)	44.3 (mean)	Healthcare workers not requiring hospitalization for COVID-19
5 [25] Brannock et al.	Clinic-based: 47,404; Model-based: 198,514	Clinic-based: 65.0% Female, 35.0% Male; Model-based: 64.6% Female, 35.4% Male	Clinic-based: 48.19 years (mean); Model-based: 47.23 years (mean)	Unvaccinated patients with COVID-19
6 [26] Ballouz et al.	1350 vaccinated	52.5% Female; 47.5% Male	48 years (median)	Individuals infected with different SARS-CoV-2 variants (Wildtype, Delta, Omicron)
7 [27] Emecen et al.	5610 vaccinated	51.8% Female, 48.2% Male	43.1 years (mean)	NR
8 [28] Ioannou et al.	198,601 vaccinated	89.1% Male, 10.9% Female	60.4 years (mean)	Unvaccinated patients with COVID-19
9 [29] Meza-Torres et al.	7,396,702 vaccinated	55.89% Female, 44.11% Male	44.5 years (median)	Unvaccinated patients with COVID-19
10 [30] Mohr et al.	419 vaccinated	84.0% Female, 15.3% Male	Age distribution: 21.5% (18–29 years), 39.9% (30–39 years), 20.3% (40–49 years), 18.4% (50–64 years)	Unvaccinated healthcare workers with COVID-19
11 [31] Taquet et al.	10,024 vaccinated individuals matched to 9,479 controls	59.4% Female, 50.6% Male	Vaccinated 57.0 years (mean), Unvaccinated 57.6 years (mean)	Unvaccinated (with influenza vaccine) patients with COVID-19
12 [32] van der Maaden et al.	9166 cases, 1698 test-negative controls, 3708 population controls	NR	NR	Unvaccinated patients with COVID-19
13 [33] Zisis et al.	1,578,719 COVID-19 patients (25,225 vaccinated)	59.8 female, 50.2% male	Vaccine group: 54.82 years (mean); No-vaccine group: 42.91 years (mean)	Unvaccinated patients with COVID-19

NR—Not Reported; List of included references [21,22,23,24,25,26,27,28,29,30,31,32,33].

**Table 3:** COVID-19 vaccination characteristics.

Reference Number and First Author	Vaccine Type *	Number of Doses	Time until Breakthrough Infection **	Follow-Up
1 [21] Al-Aly et al.	1 Janssen or 2 Pfizer/Moderna	≥2	≥14 days	6 months
2 [22] Antonelli et al.	BNT162b2, ChAdOx1 nCoV-19, mRNA-1273	2	Cases 1: Mean 73 days (median 67 days); Cases 2: Mean 51 days (median 44 days)	1 month
3 [23] Ayoubkhani et al.	74.0% received Oxford/AstraZeneca, 25.5% Pfizer/BioNTech, 0.5% Moderna	2	Median 96 days (IQR, 90–104) for double-vaccinated; Median 98 days (IQR, 89–109) for unvaccinated	≥12 weeks
4 [24] Azzolini et al.	BNT162b2	3	≥14 days	1 month
5 [25] Brannock et al.	1 Janssen or 2 Pfizer/Moderna	≥2	≥14 days	NR
6 [26] Ballouz et al.	mRNA (BNT162b2 or mRNA-1273), Adenovirus vector (JNJ-78436735)	1–3 doses	>6 months (77.6%)<6 months (22.4%)	6 months
7 [27] Emecen et al.	CoronaVac (inactivated virus), BNT162b2 (mRNA)	1 dose (96.3%), ≥2 doses (3.7%)	NR	1, 3, and 6 months
8 [28] Ioannou et al.	Moderna and Pfizer	1–2 doses	NR	3–8 months
9 [29] Meza-Torres et al.	NR	1 dose (15,832), two doses (726)	≥14 days	1–6 months
10 [30] Mohr et al.	mRNA COVID-19 vaccine	2	median of 24.1 weeks between the second vaccine dose and illness onset	6 weeks
11 [31] Taquet et al.	1 Janssen or 2 Pfizer/Moderna	1–2 doses	≥14 days	6 months
12 [32] van der Maaden et al.	1 Janssen or 2 Pfizer/Moderna	1–2 doses	>2 months	3 months
13 [33] Zisis et al.	NR	NR	NR	28 and 90 days post-COVID-19 diagnosis

NR—Not Reported; *—Janssen = Ad26.COV2.S, Pfizer = BNT162b2, Moderna = mRNA-1273; **—Time elapsed between first dose of vaccine and SARS-CoV-2 infection; List of included references [21,22,23,24,25,26,27,28,29,30,31,32,33].

**Table 4:** Outcomes and risk assessment.

Reference Number and First Author	Complications	Long COVID Risk (OR/RR/HR)	Other Risks (OR/RR/HR)
1 [21] Al-Aly et al.	ICU admissions (2.4%)	0.82 (0.80–0.85)	Mortality - 0.66 (0.58–0.74)
2 [22] Antonelli et al.	Hospitalization in frail older adults post-first dose (23%) and post-second dose (6%)	0.54 (0.36–0.80)	Frailty in older adults post-first dose (OR 1.93, 95% CI 1.50–2.48); Deprivation post-first dose (OR 1.11, 95% CI 1.01–1.23 for high deprivation)
3 [23] Ayoubkhani et al.	Long COVID symptoms: 9.5% in double-vaccinated vs. 14.6% in unvaccinated; Activity-limiting symptoms: 5.5% in double-vaccinated vs. 8.7% in unvaccinated	0.59 (0.50–0.69)	Activity-limiting symptoms: aOR 0.59 (95% CI, 0.48–0.73)
4 [24] Azzolini et al.	Long COVID prevalence: Overall 31.0%, Wave 1: 48.1%, Wave 2: 35.9%, Wave 3: 16.5%	0.29 (0.13–0.64)	Higher risk with older age (OR, 1.23), allergies (OR, 1.50), and more comorbidities (OR, 1.32)
5 [25] Brannock et al.	NR	0.70 (0.65–0.75)	Number of complications: 0.70 (0.60–0.81)
6 [26] Ballouz et al.	Long-COVID: 25.3% after Wildtype infection, 17.2% after Delta infection, 13.1% after Omicron infection	0.42 (0.24–0.68)	No clear pattern in long-COVID-related symptoms across variants; absolute risk reduction −10.6%
7 [27] Emecen et al.	ICU admissions (52.0% at 1 month, 36.2% at 3 months, 28.3% at 6 months)	0.53 (0.40–0.72)	ICU admission: 2.18 (1.51–3.14)
8 [28] Ioannou et al.	Long-COVID (13.6%)	0.78 (0.69–0.90)	Hospitalization (AOR 2.60: 2.51–2.69), mechanical ventilation (AOR 2.46: 2.26–2.69)
9 [29] Meza-Torres et al.	ICU admissions (0.7%)	0.74 (0.40–1.37)	OR 2.66 (CI 2.46–2.88) for community infection, OR 2.42 (CI 2.03–2.89) for hospital infection
10 [30] Mohr et al.	NR	0.70 (0.58–0.84)	Vaccinated patients returned to work sooner (HR 1.37, 1.04–1.79)
11 [31] Taquet et al.	NR	1.01 (0.96–1.05)	NR
12 [32] van der Maaden et al.	NR	0.90 (0.75–1.08)	Higher symptom prevalence in cases vs. controls (48.5% vs. 29.8% test-negative and 26.0% population)
13 [33] Zisis et al.	NR	0.42 (0.39–0.44)	Hypertension OR 0.33 (0.26–0.42), Respiratory symptoms OR 0.54 (0.50–0.57), Diarrhea and constipation OR 0.44 (0.40–0.49)

NR—Not Reported; ICU—Intensive Care Unit; OR—Odds Ratio; RR—Risk Ratio; HR—Hazard Ratio; List of included references [21,22,23,24,25,26,27,28,29,30,31,32,33].

## Data Availability

Not applicable.
